# Immunomodulatory effects of conditioned medium from high hydrostatic pressure-treated head and neck squamous cell carcinoma cells regarding macrophage polarization

**DOI:** 10.3389/fimmu.2026.1885288

**Published:** 2026-10-19

**Authors:** Vivica Freiin Grote, Laura Schaffran, Daniel Strüder, Julia Kristin Brach, Agmal Scherzad, Stephan Hackenberg, Rainer Bader, Anika Jonitz-Heincke

**Affiliations:** 1 Department of Orthopaedics, Biomechanics and Implant Technology Research Laboratory, Rostock University Medical Center, Rostock, Germany; 2 Department of Otorhinolaryngology, Head and Neck Surgery “Otto Körner”, Rostock University Medical Center, Rostock, Germany; 3 Department of Otorhinolaryngology, Head and Neck Surgery, University Hospital RWTH Aachen, Aachen, Germany; 4 Center for Integrated Oncology (CIO), University Hospital Rheinisch-Westfälische Technische Hochschule (RWTH) Aachen, Aachen, Germany; 5 Department of Oto-Rhino-Laryngology, Plastic, Aesthetic and Reconstructive Head and Neck Surgery, University of Würzburg, Würzburg, Germany

**Keywords:** cytokine release, head and neck cancer, high hydrostatic pressure, immune modulation, macrophage polarization, orthotopic reimplantation, tumor microenevironment

## Abstract

Surgical resection of cartilage-infiltrating head and neck squamous cell carcinoma (HNSCC) frequently results in significant tissue defects, which require reconstructive interventions. Orthotopic reimplantation of autologous laryngeal cartilage tissue could be a promising approach due to its anatomical conformity and excellent biocompatibility. High hydrostatic pressure (HHP) at 300 MPa effectively eliminates residual malignant cells while preserving the extracellular matrix and biomechanical properties of hyaline cartilage. However, HHP alters the release of tumor-derived cytokines and damage-associated molecular patterns. Given the critical role of balanced inflammatory and immunomodulatory responses in tissue integration, this *in vitro* study investigated whether factors released after HHP treatment influence macrophage polarization. Conditioned medium (CM) from HHP-treated or untreated HNSCC cell lines was used to stimulate adherent mononuclear cells for 72 hours. Unstimulated cells, M1 (GM-CSF + LPS), and M2 (M-CSF + IL-4) macrophage controls served as controls. Macrophage morphology, gene expression, and protein secretion were analyzed for M1 markers (TNF-α, IL-1β, IL-6, IL-8, IL-12 p70, IL-23, CXCL9, CXCL10) and M2 markers (IL-10, CCL17, CCL18, MMP-9). Surface markers HLA-DR, CD80, CD86, CD192 (M1-like) and CD206, CX3CR1 (M2-like) were assessed. CM from HHP-treated cells induced a heterogeneous macrophage morphology, including spindle-shaped and rounded cells. Gene expression revealed significant upregulation of IL-1β and IL-8, a trend for IL-6, and no increase in TNF-α or IL-10. At the protein level, IL-1β and IL-8 were significantly elevated, with a trend toward increased IL-6 and CXCL9, while IL-12 p70, and IL-23 remained unchanged. IL-10 levels stayed low, and no induction of CCL17, CCL18, or CCL22 was observed. Phenotypically, most cells expressed CD86 and HLA-DR, with a high proportion positive for CD192, but only a minority expressed CD80. A notable fraction retained CD206 and CX3CR1 expression regardless of stimulation. Overall, CM derived from HHP-treated HNSCC cells induced a heterogeneous, partially activated macrophage state with predominantly M1-like features rather than a distinct M1 or M2 phenotype. These findings suggest that CM may influence the inflammatory microenvironment relevant to cartilage reimplantation without promoting a strongly immunosuppressive macrophage response. Further studies using tissue models and *in vivo* validation are required to establish its relevance for tissue integration.

## 1 Introduction

Surgical tumor resection is central to curative treatment of head and neck squamous cell carcinoma (HNSCC) and other cartilage-infiltrating head and neck tumors. Depending on the tumor site and local invasion, resection may remove cartilage, bone, or soft tissue and thereby cause functional and aesthetic deficits. Relevant examples include laryngeal carcinoma requiring partial laryngectomy with thyroid or cricoid cartilage resection, as well as tumors of the nose or auricle requiring partial resection of anatomically shaped cartilage tissue. In these situations, reconstruction must restore structural support, appearance, and function, including airway patency, voice, swallowing, nasal contour, or auricular shape (1–3). Orthotopic reimplantation of autologous cartilage tissue removed because of superficial tumor infiltration or surgical safety margins could be particularly advantageous, as it preserves patient-specific anatomy and biocompatibility. However, oncological safety requires complete devitalization of residual tumor cells without compromising the integrity of the hyaline-like cartilage matrix or inducing adverse local immune effects (4).

For this purpose, high hydrostatic pressure (HHP) technology is considered as a promising tissue processing approach. HHP treatment is frequently used in food safety applications including preservation of fruit juices and jams, seafood, and ready-to-eat meat products to inactivate bacteria and enzymes while preserving color, taste and vitamin content (5–7). In addition, the physical treatment is also becoming increasingly important in the field of graft processing as it is able to gently devitalize residual cells while preserving the structure and biomechanical properties of the treated tissue (8–10). HHP is a non-thermal, physical method that allows for a standardized treatment as it transmits pressure uniformly and instantly on every cell throughout the tissue according to Pascal’s principle (9, 11).

Depending on the eukaryotic cell type and HHP protocol, different pressure thresholds typically ranging from 200 to 450 MPa for durations of 10 to 60 minutes are reported to induce irreversible cell death (9, 10, 12–14). Preliminary work indicates that although fibroblasts and chondrocytes are killed at treatment levels as low as 200 MPa (8), at least 300 MPa for ten minutes is required to completely and irreversibly devitalize HNSCC cell lines and tumor specimens while maintaining a structurally intact matrix suitable for potential reimplantation (8, 13, 15). Importantly, HHP treatment induces cell death in a pressure amplitude-dependent way, with pressures ranging from 150 MPa to around 250 MPa typically inducing apoptotic cell death, and treatment starting at 300 MPa inducing late-apoptotic or necrotic-like cell death with immunogenic signals such as calreticulin translocation, ATP and HMGB1 release (8, 13). Additionally, the HHP treatment affects the release of cancer cell-derived cytokines and chemokines that can remain in the treated tissue and may further influence cells in the tumor microenvironment (15, 16). At higher HHP levels, the concentration of Interleukin (IL)-6 and IL-8 in the supernatants were reduced compared to untreated controls (16). In contrast, Interleukin-1 (IL-1)α and IL-1β levels were elevated following HHP treatment (16).

For the safe integration of HHP-treated tissue, the inflammatory potential of these residual cytokines on macrophage polarization is of importance. As part of the innate immune system, monocyte-derived macrophages are among the first cells to migrate to the defect site (17). Macrophages significantly influence tissue integration, given that they are capable of mediating inflammation, matrix remodeling, angiogenesis, immune surveillance, and tumor progression in the graft bed and surrounding tissue (17, 18). They are highly plastic and respond to changes in the cytokine environment by altering their polarization state, which significantly influences their function (18).

For simplicity, macrophages are frequently categorized into classically activated M1 macrophages and alternatively activated M2 macrophages, sometimes subdivided into further subgroups. M1 macrophages are primarily responsible for eliminating pathogens and dead cells through phagocytosis, and contribute to anti-tumor immunity. For successful integration of HHD-treated tissue, excessive or chronic M1 macrophage-mediated inflammation should be avoided, as it could lead to tissue damage (19). Their polarization is typically induced by interferon (IFN)-γ, damage-associated molecular patterns (DAMPs) and microbial products such as lipopolysaccharide (LPS). Phenotypically, M1 macrophages are characterized by the expression of surface markers such as chemokine receptor CD192 (20), the costimulatory molecules CD86 and CD80, and a high expression of HLA-DR, reflecting their strong antigen-presenting capacity. Additionally, they are defined by the secretion of pro-inflammatory cytokines, including tumor necrosis factor (TNF)-α, IL-1β, IL-6, IL-8, IL-12, IL-23, as well as chemokine ligand (CXCL) 9, and CXCL10 (17–19, 21, 22).

Cytokines such as IL-4, IL-13, IL-10 and transforming growth factor (TGF)-β as well as immune complexes, glucocorticoids, and certain tumor-derived signals are able to induce M2 macrophage polarization (18, 19, 21). Although marker expression may differ between the various M2 subgroups, the release of anti-inflammatory cytokines IL-10 and TGF-β, as well as a high expression of the mannose receptor CD206 and the scavenger receptor CD163 on the surface of the cell membrane are considered to be the key characteristics of M2 macrophages (17, 18). They mediate anti-inflammatory or immune-regulatory functions (19), while also promoting wound healing and tissue repair (18). However, a strong M2 macrophage activity may contribute to fibrosis (17, 19). In addition, tumor-associated macrophages often exhibit an M2-like phenotype that suppresses T-cell immunity, promotes tumor growth, and metastasis (18, 19). Therefore, it is essential to investigate whether tumor cell-derived mediators released during HHP treatment influence the development of tumor-associated macrophages.

This study investigates how conditioned medium from HNSCC cells treated with HHP influences macrophage polarization. By assessing macrophage morphology, gene expression, cytokine secretion, and surface markers we aim to elucidate the mechanisms through which HHP-induced changes in cytokine milieu modulates immune responses and how it may influence tissue integration or antitumor immunity.

## 2 Materials and methods

The experimental setup for investigating the immunological response of human peripheral blood mononuclear cells (PBMCs) to conditioned medium (CM) from HHP-treated HNSCC cells is visualized in **Figure 1**.

**Figure 1:**
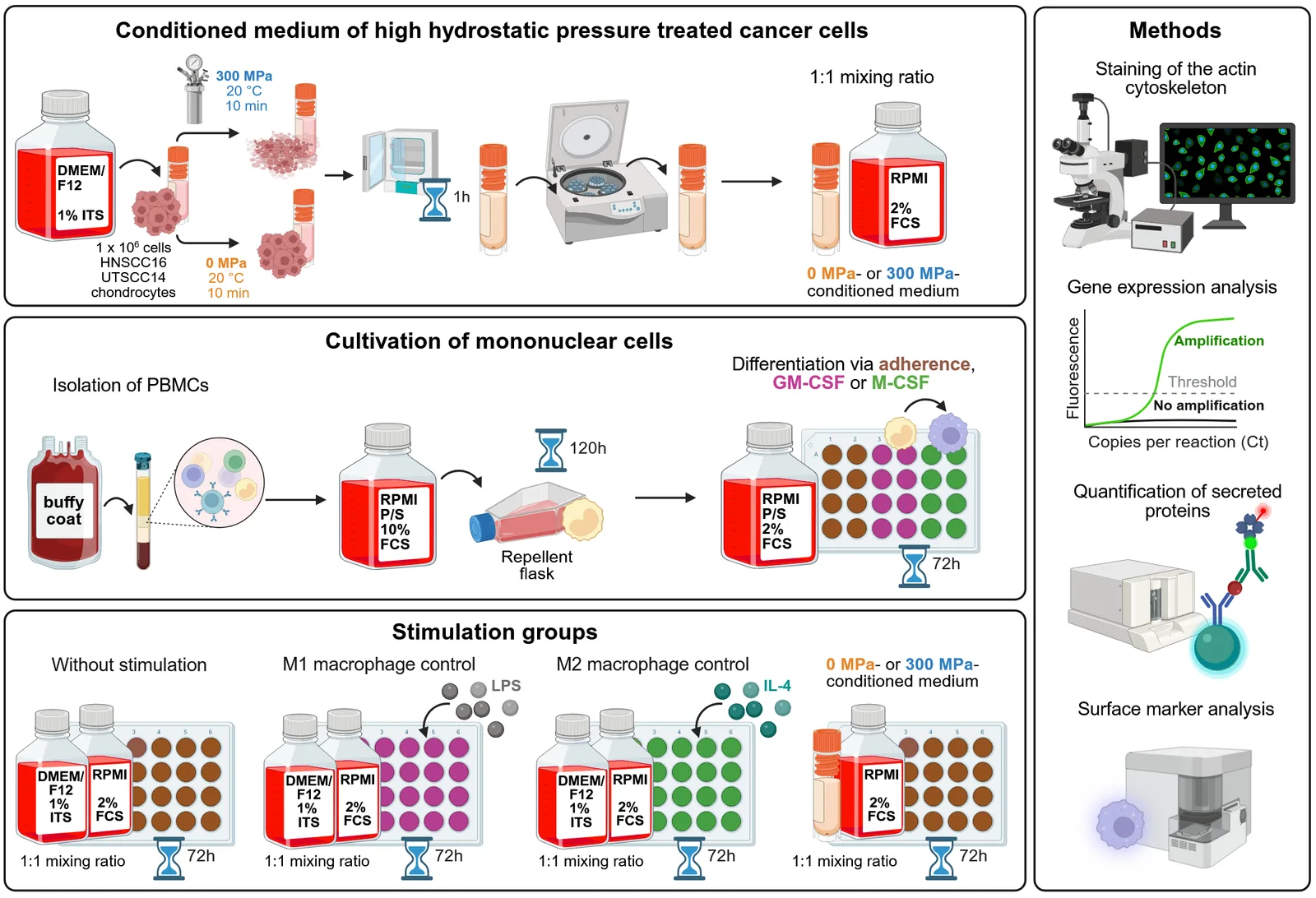
Schematic overview of the experimental workflow of the study and methods used. Created in BioRender. Jonitz-Heincke, A (2026). https://BioRender.com/0595lga.

### 2.1 HNSCC cell cultivation

Human, HPV-negative cell lines of head and neck squamous cell cancer (HNSCC) of the tongue (UTSCC14; RRID: CVCL_7810) and larynx (HNSCC16; primary tumor cell line of a patient-derived xenograft authenticated by STR profiling (23)) were used for the generation of cancer-derived CM (local ethics committee reference no. A2018-0003). HNSCC cells were cultivated in DMEM/Ham’s-F12 supplemented with 10% fetal calf serum (FCS), 2 mmol/L L-glutamine (all: Pan Biotech, Aidenbach, Germany), 1% penicillin/streptomycin (P/S), and 1% amphotericin B (all: Sigma-Aldrich, Munich, Germany) at 5% CO_2_ and 37 °C. The cells were passaged with 80% to 90% confluency.

### 2.2 Chondrocyte cultivation

For initial screening of changes in gene expression and morphology, pooled CM of HHP-treated human chondrocytes (n = 4 independent biological replicates, age: 65 ± 3 years old) were used as an additional control. For this purpose, human chondrocytes were isolated according to Jonitz et al. (24) from patients undergoing primary total knee replacement (local ethics committee reference no. A2009‐17). Isolated chondrocytes were cultivated at 37 °C, 5% CO_2_, and 21% O_2_ in Dulbecco DMEM (Gibco, ThermoFisher Scientific, Waltham, MA, USA) supplemented with 10% FCS, 1% P/S, 1% amphotericin B and 50 µg/ml ascorbic acid (all: Sigma-Aldrich, Merck, Darmstadt, Germany). For the preparation of CM, chondrocytes that had been propagated up to the third passage were utilized.

### 2.3 High hydrostatic pressure treatment and generation of conditioned medium

The generation of CM was carried out according to Brach et al. (15, 16). Briefly, 1 x 10^6^ HNSCC cells or chondrocytes were transferred in cryotubes (1.8 mL Nunc, ThermoFisher Scientific, Waltham, MA, USA), which were filled with FCS-free DMEM/Ham’s-F12 supplemented with 2 mmol/L stable L-glutamine, 1% P/S and 1% insulin-transferrin-selenium (ITS; Corning, Massachusetts, USA). Cryotubes were filled without the presence of air bubbles, sealed with Parafilm M Laboratory Film (Amcor Flexibles North America, WI, USA) and placed into a 50 mL centrifuge tube filled with water without air bubbles. The samples were treated for 10 min at 300 MPa HHP at 20 °C in a customized HHP device (Dustec Hochdrucktechnik GmbH, Wismar, Germany). The HHP treatment comprised the following pressure phases: An initial pressure built-up phase (2.5 MPa/s until 280 MPa was achieved) was followed by a lower rate of pressure increase (1 MPa/s) until the target pressure of 300 MPa was reached. After 10 min of constant 300 MPa-treatment, pressure was decreased at a rate of 2.5 MPa/s. After HHP treatment, the cells were incubated for 1 h at 37 °C with 5% CO_2_ in a humified incubator before the supernatant was collected, pooled and frozen at -80 °C until further use. Untreated, viable control samples (0 MPa) were handled accordingly.

### 2.4 Isolation & cultivation of human PBMCs

Human PBMCs were isolated from buffy coats obtained from blood donations provided by the Institute of Transfusion Medicine at Rostock University Medical Center (local ethics commission registration number: A2011-140). The isolation of PBMCs and enrichment of monocytes was performed according to previous descriptions of Klinder et al. (25). In brief, blood samples were diluted 1:1 with phosphate-buffered saline (PBS; Biochrom, Berlin, Germany). Cells were separated by density gradient centrifugation on Histopaque-1077 (Sigma-Aldrich, Munich, Germany). The resulting interphase containing PBMCs was collected and washed. For monocyte enrichment, PBMCs were seeded in cell culture flasks with cell repellent surface (Cellstar, Greiner Bio-One, Frickenhausen, Germany) (25) and cultivated in Roswell Park Memorial Institute-1640 medium (RPMI; Pan Biotech, Aidenbach, Germany) supplemented with 1% P/S, 1% amphotericin B, 2 mmol/L stable L-glutamine, 10% FCS for five days without change of medium.

### 2.5 Stimulation of adherent mononuclear cells with conditioned medium or cytokines

Monocyte-enriched PBMCs were seeded either in duplicate in 24-well plates (Primaria, Corning, MA, USA) at a density of 0.4 x 10^5^ cells per well for actin cytoskeleton staining (n = 5 independent biological replicates), gene expression analysis (n = 3–11 independent biological replicates), and protein quantification (n = 5-9 independent biological replicates), or in triplicate in 6-well plates (n = 6 independent biological replicates) (Nuclon Delta Surface, ThermoFisher Scientific, Waltham, MA, USA) at a density of 2 x 10^6^ cells per well for flow cytometry. Cells were initially cultured in RPMI supplemented with 1% P/S, 1% amphotericin B, 2 mmol/L stable L-glutamine, and 2% FCS and allowed to adhere over 72 h (26). After 72 h, the supernatant was removed and adherent mononuclear cells were stimulated with CM for an additional 72 h. For this purpose, pooled supernatants from untreated, viable cells (0 MPa-CM) or 300 MPa HHP-treated (300 MPa-CM) cells were diluted 1:1 with fresh RPMI supplemented to final concentration of 2 mmol/L stable L-glutamine, 1% P/S, 1% amphotericin B, and 2% FCS.

For control conditions, mononuclear cells were stimulated with a 1:1 mixture of fresh DMEM/F12 and RPMI supplemented with the same additives (w/o control) and either 50 ng/mL granulocyte-macrophage colony-stimulating factor (GM-CSF; ImmunoTools, Friesoythe, Germany) and 100 ng/mL lipopolysaccharide (LPS; Merck, Darmstadt, Germany) to induce M1 macrophage polarization (M1 control), or 50 ng/mL macrophage colony-stimulating factor (M-CSF; PeproTech, ThermoFisher Scientific, Waltham, MA, USA) and 20 ng/mL IL-4 (PeproTech, ThermoFisher Scientific, Waltham, MA, USA) to induce M2 macrophage polarization (M2 control).

### 2.6 Staining of the actin cytoskeleton

The staining of the cytoskeleton and nucleus was performed at room temperature (RT) and protected from light. After 72 h of stimulation, cell culture medium was removed and the cells were washed twice with phosphate-buffered saline (PBS; Biochrom AG, Berlin, Germany). Cells were then fixed by incubating with 4% paraformaldehyde (PFA, pH 7.0) for 10 min. Following fixation, the PFA solution was removed and cells were rinsed with PBS for 30 s. For membrane permeabilization, the cells were incubated with 0.05% Triton-X (Merck, Darmstadt, Germany) for 5 min. The permeabilization solution was subsequently removed and the cells were washed with PBS. Next, actin staining was performed by incubating the cells with 100 nM Acti-Stain 488 Fluorescent Phalloidin (Cytoskeleton, Denver, CO, USA) diluted in PBS for 30 min. After staining, the cells were washed three times with PBS. Nuclear staining was then carried out by incubating the cells with a 4’, 6-diamidino-2-phenylindole dihydrochloride (DAPI; Merck, Darmstadt, Germany) staining solution (1:1000 dilution in PBS) for three min. Finally, fresh PBS was added and the stained cells were stored in a refrigerator at 4 °C protected from light. Fluorescence images were acquired using a Keyence BZ-X810 fluorescence microscope (Keyence Germany, Neu-Isenburg, Germany). Images were captured at the same field of view and subsequently combined using Keyence BZ-X800 Analyzer Software to generate composite fluorescence images.

### 2.7 Gene expression analysis

In order to analyze the gene expression rate of macrophage polarization markers, quantitative real-time polymerase-chain-reaction (qPCR) was performed. Following the 72-hour stimulation period, total RNA was isolated using the innuPREP RNA mini kit 2.0 (Analytik Jena, Jena, Germany) according to the manufacturer’s protocol. Tecan Infinite 200 Pro microplate reader and NanoQuant plate (all: Tecan Group, Männedorf, Switzerland) were used to determine the RNA concentration of the samples. For all samples, 100 ng of RNA was transcribed into complementary DNA (cDNA) in a thermocycler (Analytik Jena, Jena, Germany) using the high-capacity cDNA reverse transcription kit (Applied Biosystems, Forster City, CA, USA). The following protocol was used: 10 min at 25 °C, 120 min at 37 °C, 5 min at 85 °C. Afterwards, samples were diluted with 20 µl RNase-free water.

Relative quantification of gene expression of the target genes was performed using a qTower 2.0 and innuMIX qPCR MasterMix SyGreen (both: Analytik Jena, Jena, Germany) under the following conditions: an initial activation time of 2 min at 95 °C, followed by 40 times of rotation of denaturation for 5 s at 95 °C and annealing/elongation for 25 s at 60-65 °C. The primer sequences used for the housekeeping gene (*HPRT*) and macrophage polarization-associated genes (*IL1B*, *IL6*, *IL8*, *IL10*, *TNFA*, and *MMP9*) are listed in **Table 1**. A cycle of threshold (Ct) of 32 was set as the cut-off. The relative expression of mRNA compared with the housekeeping gene *HPRT* was calculated by the equation ΔCt = Ct_gene_ – Ct*_HPRT_*. The relative expression of target mRNA of the samples was calculated using the 2^(-ΔΔCt)^ method.

**Table 1:** Primer sequences used for quantitative real-time PCR.

Gene	Forward primer (5’ - 3’)	Reverse primer (5’ - 3’)
Hypoxanthine-guanine phosphoribosyltransferase (*HPRT*)	CCCTGGCGTCGTGATTAGTG	TCGAGCAAGACGTTCAGTCC
Interleukin-1β (*IL1B*)	TACTCACTTAAAGCCCGCCT	ATGTGGGAGCGAATGACAGA
Interleukin-6 (*IL6*)	TGGATTCAATGAGGAGACTTGCC	CTGGCATTTGTGGTTGGGTC
Interleukin-8 (*IL8*)	TCTGTGTGAAGGTGCAGTTTTG	ATTTCTGTGTTGGCGCAGTG
Interleukin-10 (*IL10*)	AGCTGGACAACTTGTTGTTAAAGG	CAGGGAAGAAATCGATGACAGC
Tumor necrosis factor-α (*TNFA*)	GTTGTAGCAAACCCTCAAGCTG	GAGGTACAGGCCCTCTGATG
Matrix metallopeptidase-9 (*MMP9*)	ACGCAGACATCGTCATCCAG	AACCGAGTTGGAACCACGAC

### 2.8 Quantification of secreted proteins in the supernatants

In order to normalize target protein levels to total protein content in each sample, total protein was quantified using the Qubit Protein Assay Kit and the Qubit 3.0 Fluorometer (both: Thermo Fisher Scientific, Waltham, MA, USA) according to the manufacturer’s instructions.

#### 2.8.1 Cytokine multiplex analysis

Protein content of CCL17, CCL18, CCL22, CXCL9, CXCL10, IL-1β, IL-6, IL-8, IL-12 p70, IL-23, matrix metalloproteinase (MMP)-9 and TNF-α in the supernatants of the stimulated mononuclear cells after 72 h was analyzed using a customized Luminex human discovery multiplex assay (Human premixed Multi-Analyte-Kit, #LXSAHM, R&D Systems, Minneapolis, MN, USA) according to the manufacturer’s instructions. All incubation steps were performed at RT on an orbital shaker protected from light. In short, standards and supernatant samples were diluted (1:1 with Calibrator Diluent RD6-52) and incubated with magnetic beads pre-coated with analyte-specific antibodies and a specific fluorescent color coding for 2 hours. Following several washing steps using a hand-held microplate magnet, a biotinylated antibody solution was added and incubated for one hour. Unbound antibodies were removed via further washing steps. Next, streptavidin-phycoerythrin (PE) conjugate was added and incubated for 30 min. Unbound streptavidin-PE was removed and samples were measured on a Luminex 100/200 using xPONENT 3.1.871.0 software. For the standard curve fitting analysis, a four-parameter logistic (4-PL) nonlinear regression model was employed (GraphPad PRISM software, version 9.5.1, San Diego, CA, USA). To account for baseline protein levels, the protein content of the medium blanks (0 MPa-CM blank, 300 MPaCM blank and fresh medium blank) was deducted from the respective samples. Negative values obtained after blank subtraction were considered below the limit of detection and imputed as 0.

#### 2.8.2 IL-1β ELISA

To further analyze the amount of IL-1β secreted by stimulated macrophages and to account for the cytokine’s half-life in the blanks of the CM derived from HHP-treated HNSCC cells, an additional ELISA was performed. In this assay, blanks were incubated in cell-free wells under the same conditions as the stimulated cells during the 72-hour stimulation period. Subsequently, the IL-1β concentration in the samples and blanks was determined using an IL-1β ELISA (Human IL-1-beta ELISA Ready-SET-Go!; ThermoFisher Scientific, Waltham, MA, USA). Absorbance was measured at 405 nm using a Tecan Infinite 200 Pro microplate reader (Tecan Group AG, Männedorf, Switzerland). Cytokine concentrations were calculated based on a standard curve. The IL-1β values measured in the incubated blanks were subsequently subtracted from those obtained from the stimulated samples.

### 2.9 Flow cytometry and gating strategy

Following stimulation, adherent macrophages (n = 6 independent biological replicates) were detached by incubation on ice for 20 min, followed by mechanical scraping and resuspension by pipetting. An intracellular pan-macrophage marker and several surface markers for macrophage polarization were analyzed by flow cytometry using a Cytek Aurora and the acquisition software SpectroFlo version 2.2.0.3 (both: Cytek Biosciences, Fremont, CA, USA). Two separate multicolor panels were used to assess either M1 or M2 macrophage polarization markers. All staining procedures were performed protected from light. For each panel, 1 x 10^6^ cells were stained using the Zombie NIR Fixable Viability Kit (1:1000 in 100 µl PBS; BioLegend, San Diego, CA, USA) for 30 min at RT, followed by two washing steps with staining buffer (PBS containing 2 mM ethylenediaminetetraacetic acid and 2% bovine serum albumin). Cells were then incubated with True-Stain Monocyte Blocker and Human TruStain FcX (both: BioLegend, San Diego, CA, USA) for 8 min at RT to prevent nonspecific antibody binding. The subsequent extracellular staining was performed on ice for 15 min.

The M1 macrophage panel included the following fluorochrome-conjugated antibodies (all: BioLegend, San Diego, CA, USA): Brilliant Violet 510 anti-human HLA-DR (clone L243), Alexa Fluor 647 anti-human CD80 (clone 2D10), PE/Cyanine7 anti-human CD86 (clone BU63), and Brilliant Violet 421 anti-human CD192 (CCR2; clone K036C2). The M2 marker Panel consisted of the following antibodies: Brilliant Violet 510 anti-human HLA-DR (clone L243), APC anti-human CD163 (GH1/61 clone), Brilliant Violet 421 anti-human CD206 (15–2 clone), and PE/Cyanine7 anti-human CX3CR1 (2A9–1 clone).

After two washing steps, cells were fixed with 4% PFA for 20 min at RT. Following fixation, cells were washed and permeabilized by resuspending them twice in Intracellular Staining Permeabilization Wash Buffer (BioLegend, San Diego, CA, USA) and centrifuged at 350 x g for 5 min. Intracellular staining was performed using PE anti-human CD68 (Y1/82A clone; BioLegend, San Diego, CA, USA) for 20 min at RT. Cells were washed again, resuspended in staining buffer, and stored at 4 °C protected from light until acquisition. Flow cytometry data were analyzed using FlowJo (version 10.7.1; FlowJo LLC, Ashland, OR, USA). The gating strategy used for macrophage identification and marker analysis is shown in the **Supplementary Figure 1**.

### 2.10 Graphical illustration and statistical analysis

If not otherwise stated, data are presented as individual values (one data point per donor) with median and interquartile ranges. Graphical illustration and statistical analysis of the data were performed using GraphPad PRISM software, version 9.5.1 (GraphPad Software, San Diego, CA, USA). A p-value of less than 0.05 was defined as statistically significant. Outliers were identified using the ROUT method (Q = 1%). To account for the sample size and to ensure a consistent statistical approach across all datasets, group comparisons were performed using non-parametric Kruskal-Wallis test followed by Dunn’s multiple comparison test, irrespective of the outcome of the normality assessment.

## 3 Results

### 3.1 Assessment of macrophage morphology upon different stimulation conditions

To investigate whether mediators released by HHP-treated or untreated HNSCC cells influence macrophage polarization, we first assessed morphological changes. For this purpose, cell nuclei and actin cytoskeleton were stained in unstimulated samples (**Figure 2A**), cells stimulated with CM derived from 300 MPa-treated chondrocytes (**Figure 2B**), M1 macrophage controls (**Figure 2C**), M2 macrophage controls (**Figure 2D**), and cells treated with CM derived from untreated (0 MPa; **Figures 2E, G**) or HHP-treated (300 MPa; **Figures 2F, H**) HNSCC cell lines HNSCC16 (**Figures 2E, F**) and UTSCC14 (**Figures 2G, H**).

**Figure 2:**
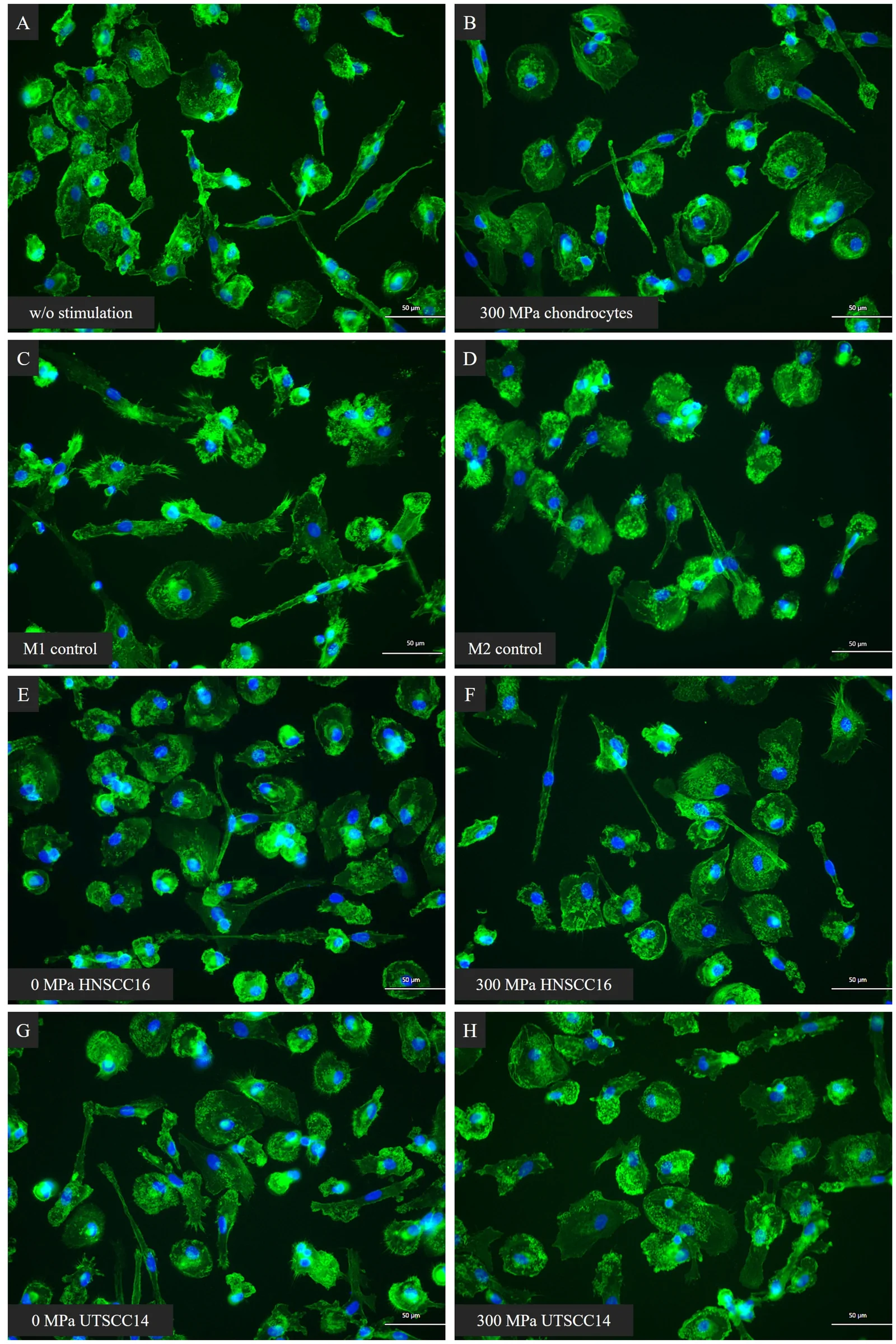
No distinct shift in morphology of adherent mononuclear cells is observed following stimulation with conditioned medium (CM) derived from high hydrostatic pressure (HHP)-treated head and neck squamous cell cancer (HNSCC). The actin cytoskeleton staining of macrophages (n = 5 independent biological replicates) was performed after 72 hours of cultivation with fresh medium [w/o stimulation; **(A)**] or CM of 300 MPa HHP-treated chondrocytes **(B)**, serving as negative controls. Macrophages stimulated with GM-CSF and LPS [M1 control, **(C)**] or M-CSF and IL-4 [M2 control, **(D)**] served as positive controls and were compared with cells treated with CM derived from untreated [0 MPa; **(E, G)**] or HHP-treated (300 MPa; F, H) HNSCC cell line HNSCC16 **(E, F)** or UTSCC14 **(G, H)**. Across all experimental groups, cells displayed a heterogeneous morphology consisting predominantly of larger egg-shaped or irregular-shaped cells with membrane ruffling, elongated spindle-like cells, and a few smaller round cells, indicating a heterogeneous cell population. No distinct morphological differences between stimulation conditions or control groups were observed. The actin cytoskeleton was stained with phalloidin (green fluorescence). The nuclei were counterstained with DAPI (blue fluorescence). Fluorescence images were acquired using a Keyence BZ-X810 fluorescence microscope and combined using Keyence BZ-X800 Analyzer Software to generate composite fluorescence images (scale bar 50 µm).

After 72 hours of stimulation, macrophages with heterogeneous morphologies were observed across all experimental groups (**Figure 2**), displaying an overall larger and more distinct appearance compared to cells after 24 hours of incubation (**Supplementary Figure 2**). However, a few smaller and round cells remained in all experimental groups, suggesting a mixed culture containing immature macrophages or other leukocytes. While predominantly larger, egg-shaped or irregular-shaped cells with membrane ruffling were observed following treatment with CM derived from HNSCC cells, independent of cell line and HHP treatment, elongated, spindle like cells were also commonly found. This heterogeneous appearance was consistently detected irrespective of the applied stimulation condition, including M1 and M2 macrophage controls. Notably, M1 control cells showed a morphology that tends to be more elongated with large round or irregular shaped cell types. Nevertheless, due to the heterogeneity and overlapping structural characteristics, based solely on morphological criteria, a clear classification of the cells into M1 or M2 macrophage phenotypes was not possible.

These observations indicate that macrophage morphology at the 72-hour time point reflects a heterogeneous cellular response rather than a distinct morphological switch between polarization states. Consequently morphological assessment by microscopy alone was considered insufficient to discriminate between M1- or M2-like phenotypes and was therefore complemented by gene expression, protein, and surface marker analyses.

### 3.2 Conditioned medium derived from high hydrostatic pressure-treated head and neck squamous cell cancer induces selected inflammatory gene expression in adherent mononuclear cells

To assess the extent to which mediators contained in cancer-derived conditioned media may influence the macrophage polarization in human adherent mononuclear cells, gene expression analysis of M1 macrophage and M2 macrophage polarization markers was conducted. Relative mRNA expression levels were calculated using the 2^(-ΔΔCt)^ method and presented as fold change relative to the unstimulated control (**Figure 3**).

**Figure 3:**
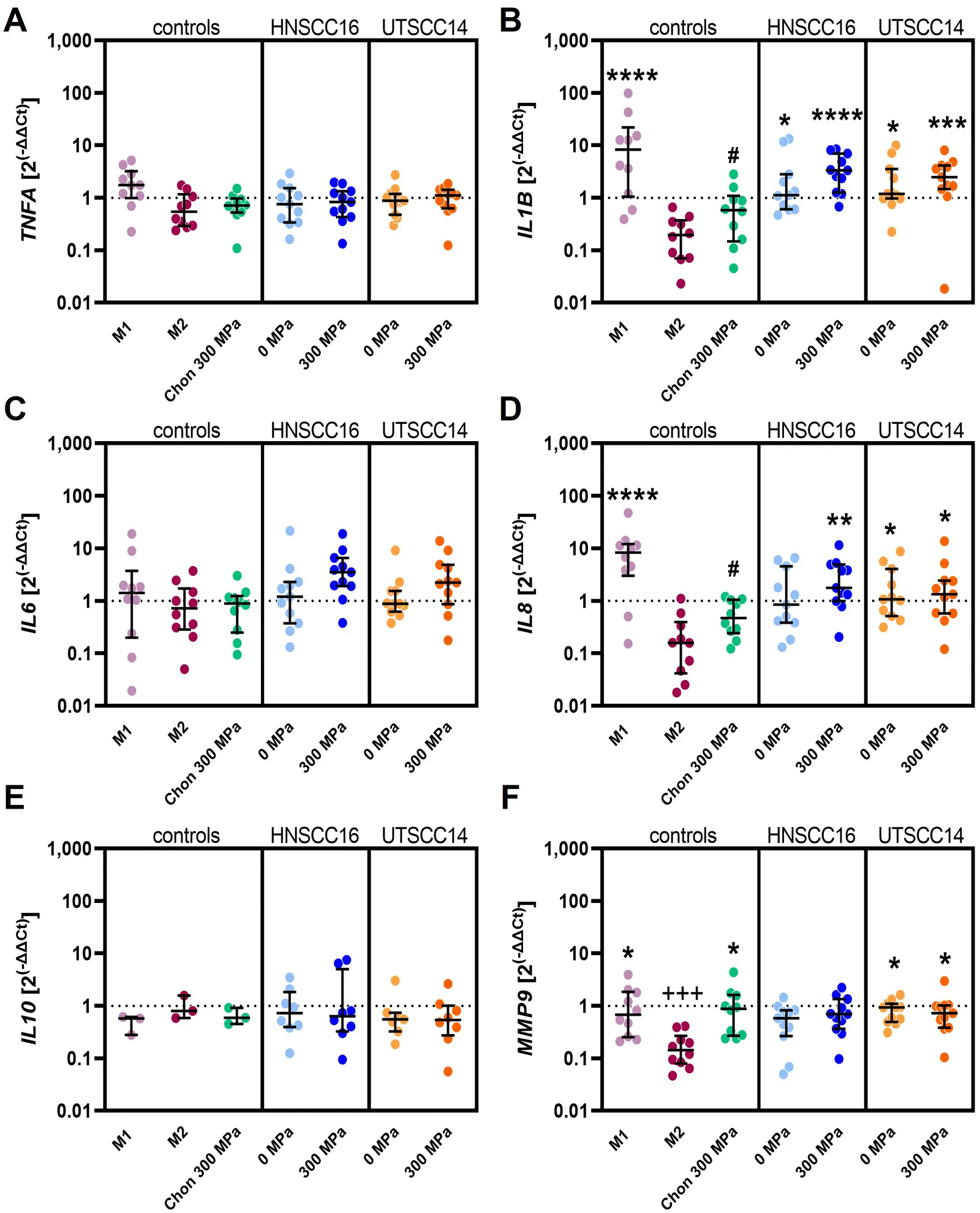
Stimulation with conditioned medium (CM) of high hydrostatic pressure (HHP)-treated head and neck squamous cell carcinoma (HNSCC) cell lines significantly elevates gene expression of *Interleukin (IL) 1B* and *IL8* in human macrophages. PBMCs were stimulated for 72 hours with CM derived from untreated (0 MPa) or 300 MPa HHP-treated HNSCC cells (HNSCC16 or UTSCC14). GM-CSF/LPS and M-CSF/IL-4 were used as positive controls for M1 and M2 macrophage polarization, respectively, while fresh medium served as an unstimulated control (w/o). Gene expression of the M1 macrophage markers *TNFA* **(A)**, *IL1B* **(B)**, *IL6* **(C)**, *IL8* **(D)** and the M2 macrophage markers *IL10* **(E)**, and *MMP9* **(F)** was analyzed by qPCR. Relative mRNA expression levels were calculated using the 2^(-ΔΔCt)^ method, with *HPRT* as the housekeeping gene, and data are presented as fold change relative to the unstimulated control (set to 1 and illustrated as dotted lines). Data are shown as median and interquartile range. Statistical significance was determined using the Kruskal-Wallis test with Dunn’s multiple comparisons test for (n = 3–11 independent biological replicates; * vs. M2 control, p < 0.05; ** vs. M2 control, p < 0.01; *** vs. M2 control, p < 0.001; **** vs. M2 control, p < 0.0001; ^#^ vs. M1 control, p < 0.05; ^+++^ vs. w/o stimulation, p < 0.001).

Compared to the M2 macrophage control samples, gene expression of *IL1B* (**Figure 3B**) and *IL8* (**Figure 3D**) were significantly elevated in M1 macrophage control cells (*IL1B*: p < 0.0001; *IL8*: p < 0.0001), as well as in cells stimulated with 300 MPa-CM of HNSCC16 (*IL1B*: p < 0.0001; *IL8*: p = 0.0031) and UTSCC14 cells (*IL1B*: p = 0.0007; *IL8*: p = 0.0354). Stimulation with 0 MPa-CM from vital HNSCC16 and UTSCC14 cells also led to a significant increase in the expression of both genes compared with M2 control cells. However, the magnitude of this increase was lower than that observed after 300 MPa-CM treatment. In contrast, CM derived from 300 MPa-treated chondrocytes did not induce *IL1B* and *IL8* expression, but instead significantly reduced gene expression compared to M1 control samples (*IL1B:* p = 0.0106; *IL8: p = 0.0191*). A similar tendency was observed for *IL6* (**Figure 3C**), for which expression was increased following stimulation with CM derived from 300 MPa-treated HNSCC cells, although the differences did not reach statistical significance. Relative gene expression of *TNFA* (**Figure 3A**) was highest in the M1 macrophage control group but did not differ significantly between stimulation conditions. Thus, the transcriptional response to HHP-treated HNSCC-derived CM was dominated by selected inflammatory genes rather than by a uniform induction of all analyzed M1-associated markers.

For the M2 macrophage marker *IL10* (**Figure 3E**), no statistically significant differences were observed across the different cultivation conditions. Notably, *IL10* gene expression analysis across all control groups, including the M2 macrophage control, revealed generally high Ct values, with some exceeding the cut-off threshold of 32. Furthermore, M2 macrophage control samples showed decreased relative mRNA expression levels of *MMP9* (**Figure 3F**) compared to all other stimulation groups. However, no statistically significant difference was observed between the M2 macrophage control samples and the samples stimulated with 0 MPa- or 300 MPa-CM derived from HNSCC16 cells.

Overall, the gene expression data suggest that CM derived from HHP-treated HNSCC cells induces selected inflammatory genes, particularly *IL1B* and *IL8*. However, the absence of an increase in all classical M1-associated genes indicate that the response cannot be interpreted as a conversion to a complete classical M1 macrophage phenotype. To further verify these assumptions, protein levels of the above-mentioned as well as additional macrophage markers were analyzed.

### 3.3 Influence of conditioned media on the inflammatory mediator profile of human macrophages

In addition to quantifying the mRNA expression of TNF-α, IL-1β, IL-6, IL-8, IL-10, and MMP-9, analyzing secreted proteins in the macrophage supernatant is essential for obtaining a comprehensive picture of the functional immune response. While mRNA analysis was restricted to these specific targets, protein quantification encompassed the same markers as well as additional factors: IL-12 p70, IL-23, CXCL9, CXCL10, CCL17, CCL18, and CCL22. These factors play a critical role in polarizing T-cell responses, recruiting specific immune cell populations, and distinguishing between pro- and anti-inflammatory macrophage phenotypes (18). Consequently, the combined analysis of transcript and protein levels allows for conclusions regarding both post-transcriptional regulation and the actual immunomodulatory function of the macrophages.

The secretion of pro-inflammatory cytokines and chemokines by human adherent mononuclear cells under different stimulation conditions for 72 hours was analyzed by protein multiplex analysis, respective blanks (**Supplementary Table 1**) were deducted, and target protein content was then normalized to total protein content (**Figures 4**, **5A, B**, **6**).

**Figure 4:**
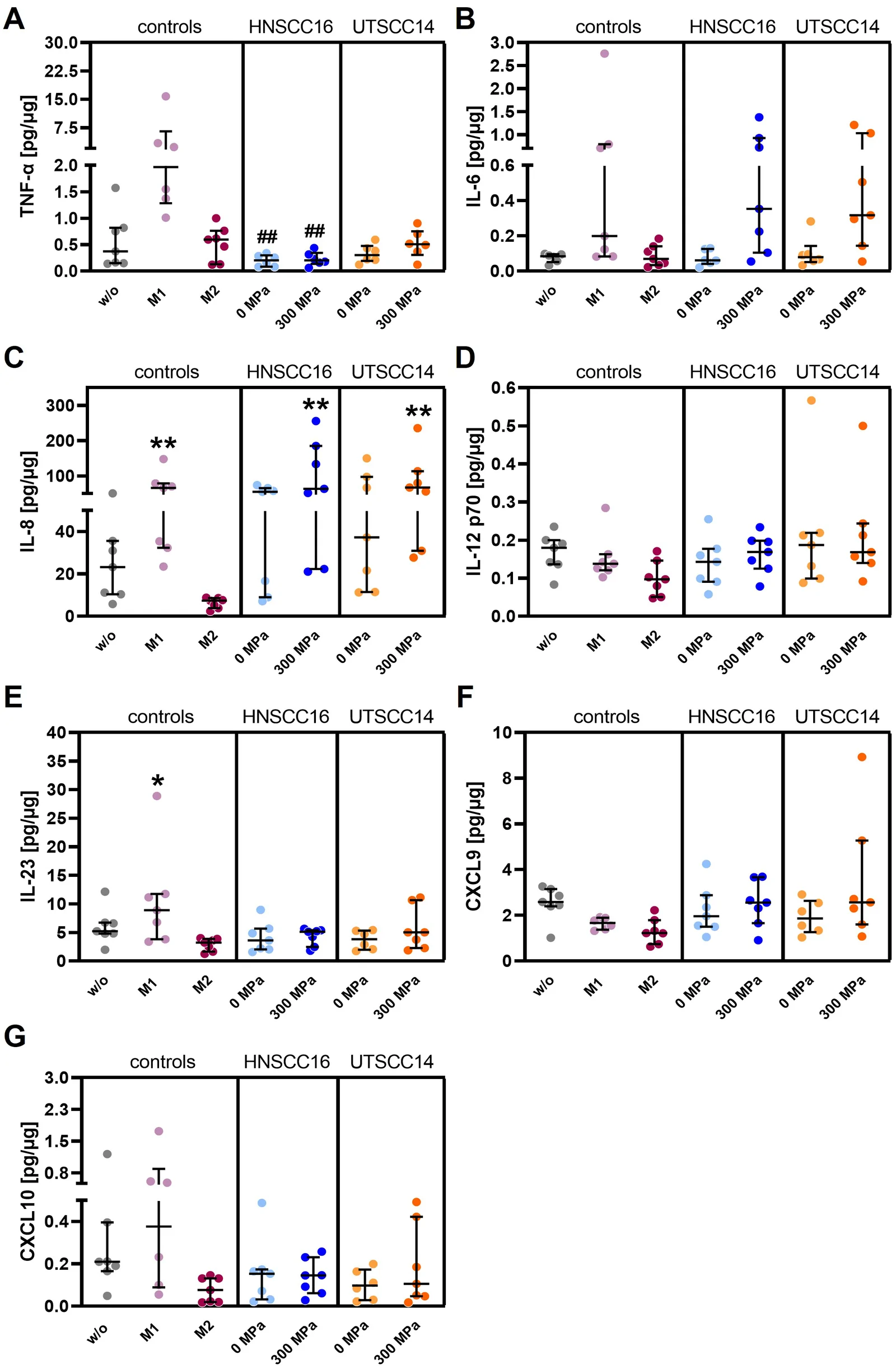
Exposure to conditioned medium (CM) from high hydrostatic pressure (HHP)-treated cancer cells resulted in an increased secretion of IL-8 and CXCL9 by human macrophages compared to M2 macrophage control samples. Human adherent mononuclear cells were stimulated for 72 hours with CM derived from untreated (0 MPa) or 300 MPa HHP-treated head and neck squamous cell cancer (HNSCC) cells (HNSCC16 or UTSCC14). GM-CSF/LPS and M-CSF/IL-4 were used as positive controls for M1 and M2 macrophage polarization, respectively, while fresh medium served as an unstimulated control (w/o). Protein content of the M1 macrophage markers TNF-α **(A)**, IL-6 **(B)**, IL-8 **(C)**, IL-12 p70 **(D)**, IL-23 **(E)**, CXCL9 **(F)**, and CXCL10 **(G)** in the supernatants was quantified by a customized Luminex discovery multiplex assay. To account for baseline protein content, values obtained from blank samples were subtracted, and cytokine concentrations were normalized to the total protein content of the respective samples, as determined by Qubit protein assay. Data are shown as median and interquartile range. Statistical significance was determined using the Kruskal-Wallis test with Dunn’s multiple comparisons test (n = 6–7 independent biological replicates; * vs. M2 control, p < 0.05; ** vs. M2 control, p < 0.01; ^##^ vs. M1 control, p < 0.01).

**Figure 5:**
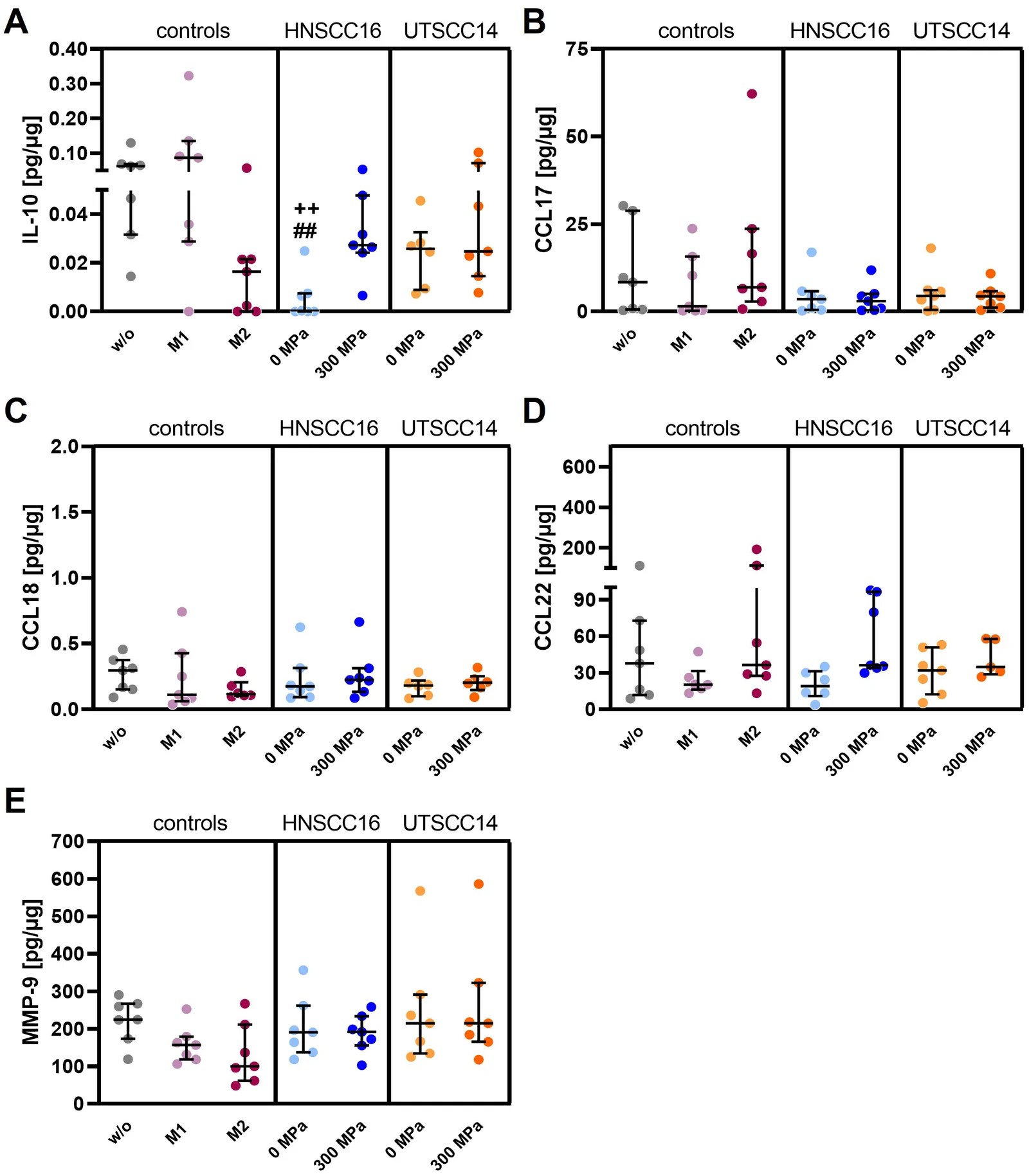
No significant differences in protein secretion of M2 macrophage markers were observed after stimulating human adherent mononuclear cells with conditioned medium (CM) of high hydrostatic pressure (HHP)-treated cancer cells. Human macrophages were stimulated for 72 hours with CM derived from untreated (0 MPa) or 300 MPa HHP-treated head and neck squamous cell cancer (HNSCC) cells (HNSCC16 or UTSCC14). GM-CSF/LPS and M-CSF/IL-4 were used as positive controls for M1 and M2 macrophage polarization, respectively, while fresh medium served as an unstimulated control (w/o). Protein content of the M2 macrophage markers IL-10 **(A)**, CCL17 **(B)**, CCL18 **(C)**, CCL22 **(D)**, and MMP-9 **(E)** in the supernatants was quantified by a customized Luminex discovery multiplex assay. To account for baseline protein content, values obtained from blank samples were subtracted, and cytokine concentrations were normalized to the total protein content of the respective samples, as determined by Qubit protein assay. Data are shown as median and interquartile range. Statistical significance was determined using the Kruskal-Wallis test with Dunn’s multiple comparisons test (n = 5–7 independent biological replicates; ^++^ vs. w/o stimulation, p < 0.01; ^##^ vs. M1 control, p < 0.01).

**Figure 6:**
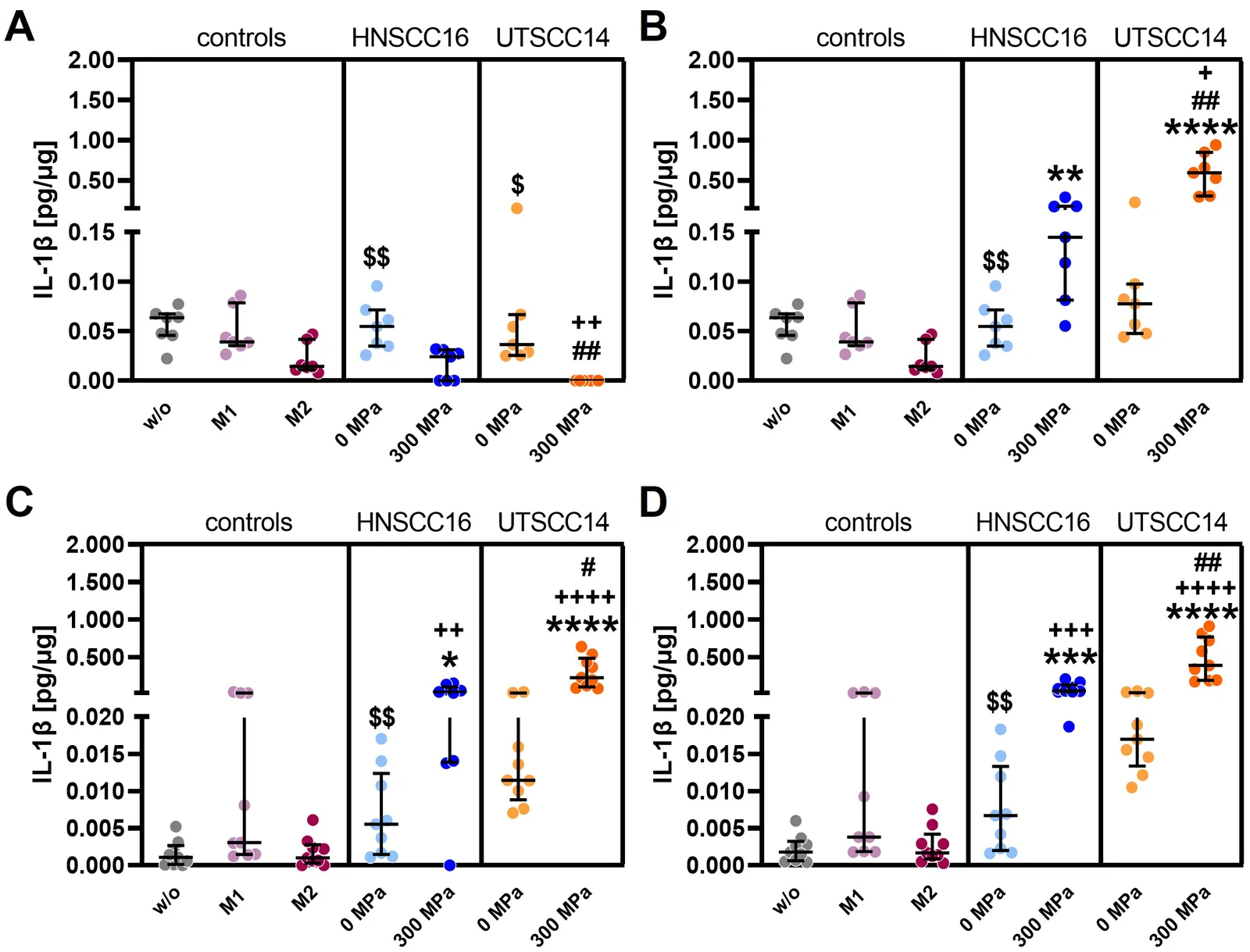
Assessment of IL-1β protein content per total protein in supernatants of human macrophages after 72 hours with different cultivation conditions. Human adherent mononuclear cells were stimulated for 72 h with conditioned media (CM) derived from untreated (0 MPa) or 300 MPa HHP-treated head and neck squamous cell cancer (HNSCC) cells (HNSCC16 or UTSCC14). GM-CSF/LPS and M-CSF/IL-4 were used as positive controls for M1 and M2 macrophage polarization, respectively, while fresh medium served as an unstimulated control (w/o). Protein content of the M1 macrophage markers IL-1β in the supernatants was quantified by a customized Luminex discovery multiplex assay **(A, B)** or by an ELISA **(C, D)**. To account for baseline protein content, values obtained from blank samples without further cultivation **(A)** or with cell-free cultivation over the 72-hour stimulation period were subtracted **(A, C)** and compared to unchanged protein values **(B, D)**. The cytokine concentrations were normalized to the total protein content of the respective samples, as determined by Qubit protein assay. Data are shown as median and interquartile range. Statistical significance was determined using the Kruskal-Wallis test with Dunn’s multiple comparisons test for non-normally distributed data (n = 7–9 biological replicates; * vs. M2 control, p < 0.05; ** vs. M2 control, p < 0.01; *** vs. M2 control, p < 0.001; **** vs. M2 control, p < 0.0001; ^#^ vs. M1 control, p < 0.05; ^##^ vs. M1 control, p < 0.01; ^+^ vs. w/o stimulation, p < 0.05; ^++^ vs. w/o stimulation, p < 0.01; ^+++^ vs. w/o stimulation, p < 0.001; ^++++^ vs. w/o stimulation, p < 0.0001; ^$^ vs. 300 MPa-CM of UTSCC14, p < 0.05; ^$$^ vs. 300 MPa-CM of UTSCC14, p < 0.01);.

The protein data supported the presence of a selective inflammatory response following 300 MPa-CM-treatment. The IL-8 protein levels (**Figure 4C**) in the supernatants of the M1 control macrophages (median: 66.30 pg/µg; vs. M2 control p = 0.0074), as well as macrophages stimulated with 300 MPa-CM (HNSCC16: 63.71 pg/µg, vs. M2 control p = 0.0039; UTSCC14: 67.36 pg/µg, vs. M2 control p = 0.0023), were significantly increased compared to M2 macrophage controls (7.398 pg/µg). Although the IL-8 content per total protein content of cells stimulated with 0 MPa-CM were also elevated, the differences did not reach statistical significance. Similarly, the IL-6 protein content per total protein (**Figure 4B**) was highest in the supernatants from macrophages stimulated with 300 MPa-CM (HNSCC16: 0.3530 pg/µg; UTSCC14: 0.3171 pg/µg) and even higher protein levels than IL-6 protein content per total protein of the M1 control cells (median 0.1994 pg/µg). However, these findings were not statistically significant.

Further, the proportion of TNF-α relative to total protein (**Figure 4A**) was not increased following stimulation with UTSCC14-derived CM (0 MPa-CM: 0.3036 pg/µg; 300 MPa-CM: 0.5073 pg/µg) and was even significantly reduced following stimulation with HNSCC16-derived CM compared with M1 macrophage control samples (0 MPa-CM: 0.2027 pg/µg; vs. M1 control p = 0.0016; 300 MPa-CM: 0.2277 pg/µg; vs. M1 control p = 0.0062). Likewise, 300 MPa-CM did not significantly influence the protein content per total protein of the additional M1-markers IL-12 p70 (**Figure 4C**), IL-23 (**Figure 4E**), or CXCL10 (**Figure 4G**). Regarding CXCL9 protein content (**Figure 4F**), a trend towards elevated amounts per total protein were determined for macrophages stimulated with CM of 300 MPa-treated UTSCC14 cells (median: 2.567 pg/µg) compared to the M2 macrophage control (median: 1.227 pg/ug). A similar median protein content was observed in cells treated with CM from 300 MPa-treated HNSCC16 (median: 2.563 pg/µg) and in unstimulated controls (median: 2.587 pg/µg), however, with no statistical significance. Notably, even though supernatants of M1 macrophage control cells contained generally higher amounts of the observed M1-markers, only the protein content per total protein for IL-23 of the M1 control samples (median: 8.902 pg/µg) showed significantly higher values compared to the M2 macrophage control samples (median: 3.217; p = 0.0366).

Regarding the protein content of mediators associated with M2 macrophages (**Figures 5A–E**), no statistically significant differences were determined between the stimulation groups and controls for the CCL17 (**Figure 5B**), CCL18 (**Figure 5C**), CCL22 (**Figure 5D**) and MMP-9 (**Figure 5E**) protein levels per total protein. As depicted in **Figure 5A**, stimulation with 300 MPa-CM did not significantly reduce IL-10 secretion, however, supernatants of cells treated with CM of 0 MPa-treated HNSCC16 contained significantly lower IL-10 per total protein (median: 0.0003275 pg/µg) compared to the unstimulated samples (median: 0.06243 pg/µg, p = 0.0095) as well as the supernatants of the M1 macrophage control cells (median: 0.08696 pg/µg, p = 0.0092). The median of the IL-10 content per total protein of the M2 macrophage control cells (median: 0.01644 pg/µg) was the second lowest of all experimental groups, but the difference was not statistically significant.

#### 3.3.1 IL-1β synthesis requires specific consideration because of its presence in CM blanks

While the protein levels for the other target proteins were largely insignificant in the CM blanks derived from 300 MPa HHP-treated HNSCC cells used for macrophage stimulation, the initial Luminex analysis revealed that the concentration of IL-1β, especially in 300 MPa-CM of UTSCC14 cells, was sufficiently high that (**Supplementary Table 1**), upon subtraction of this baseline, several samples yielded mathematically negative values after the 72-hour stimulation period and were set to 0 (median HNSCC16: 0.02436 pg/µg; median UTSCC14: 0 pg/µg) (**Figure 6A**). However, without subtraction of the blank values, but with IL-1β-content present in the supernatant normalized to total protein content, macrophages stimulated with CM derived of 300 MPa-treated HNSCC cells exhibited highest IL-1β levels (median HNSCC16: 0.1448 pg/µg; median UTSCC14: 0.5939 pg/µg) that were significantly higher than the content of M2 macrophage control cells (median M2: 0.01458 pg/µg; vs. 300 MPa HNSCC16 p = 0.0031; vs. 300 MPa UTSCC14 p <0.0001), the unstimulated control (vs. 300 MPa UTSCC14, p = 0.0255), as well as the M1 control samples (vs. 300 MPa UTSCC14, p = 0.0041) (**Figure 6B**).

To assess how much the initially added IL-1β content of the HHP-CM decreased over the 72-hour stimulation period, attributable to its limited half-life, blanks of the HHP-CM were incubated under the same conditions as the stimulated samples, but without cells, and the IL-1β concentration was determined by ELISA. IL-1β levels in incubated HHP-CM blanks were measured by ELISA and used to correct the stimulated samples by subtracting the corresponding IL-1β blank values (**Figure 6C**), thereby enabling a more accurate estimation of cell-derived IL-1β production. Statistically significant elevated IL-1β levels were observed in the samples stimulated with 300 MPa-CM of UTSCC14 cells (median: 0.2262 pg/µg) compared to unstimulated samples (median: 0.001072 pg/µg, p <0.0001), M2 control (median: 0.001001 pg/µg, p <0.0001), M1 control (median: 0.003072 pg/µg, p = 0.0144) as well as to samples treated with HNSCC16-derived 0 MPa-CM (0 MPa: 0.005541 pg/µg, p = 0.0067). Similarly, a statistically significant increase in the IL-1β protein content relative to total protein was observed in the supernatants from macrophages stimulated with CM derived from 300 MPa-treated HNSCC16 cells (median: 0.03521 pg/µg) compared to supernatants of unstimulated macrophages (p = 0.0085) as well as M2 control samples (p = 0.0141).

**Figure 6D** depicts the IL-1β concentration normalized to the total protein content without deduction of respective blank values that were assessed using IL-1β-ELISA. Similar to the Luminex measurement of IL-1β levels without blank deduction, samples treated with 300 MPa-CM contained highest amount of IL-1β per total protein (HNSCC16: 0.05009 pg/µg; UTSCC14: 0.3880 pg/µg) with significant differences compared to unstimulated control samples (median: 0.001785 pg/µg; vs. HNSCC16 p = 0.0005; vs. UTSCC14 p < 0.0001), M2 macrophage control (0.001667 pg/µg; vs. HNSCC16 p = 0.0008; vs. UTSCC14 p < 0.0001), and M1 macrophage control (median: 0.003820 pg/µg; vs. UTSCC14 p = 0.0067). Notably, IL-1β levels measured by ELISA were substantially lower than those obtained with the Luminex assay, except for the UTSCC14-derived 300 MPa-CM samples, where protein levels were nearly identical between the two methods.

Overall, the protein analyses demonstrate that HHP-treated HNSCC-derived CM induces a selective inflammatory mediator profile characterized most consistently by increased IL-8 and IL-1β. At the same time, several classical inflammatory mediators, including TNF-α and IL-12 p70, were not significantly increased. The response therefore represents partial inflammatory activation rather than a uniform classical M1 polarization.

### 3.4 Surface marker expression of macrophage polarization is influenced by cancer cell-derived conditioned medium

For the subsequent experiments, macrophages were stimulated for flow cytometry analysis using CM derived from a single tumor cell line. This decision was based on previous findings showing that macrophages exhibited very similar responses to CM from both HNSCC16 and UTSCC14 cells. To avoid redundancy and reduce experimental complexity, one representative cell line was selected. The patient-derived xenograft primary tumor cell line HNSCC16 was chosen due to its high clinical relevance. The percentage of macrophages expressing specific surface markers was then determined by flow cytometry (**Figure 7**).

**Figure 7:**
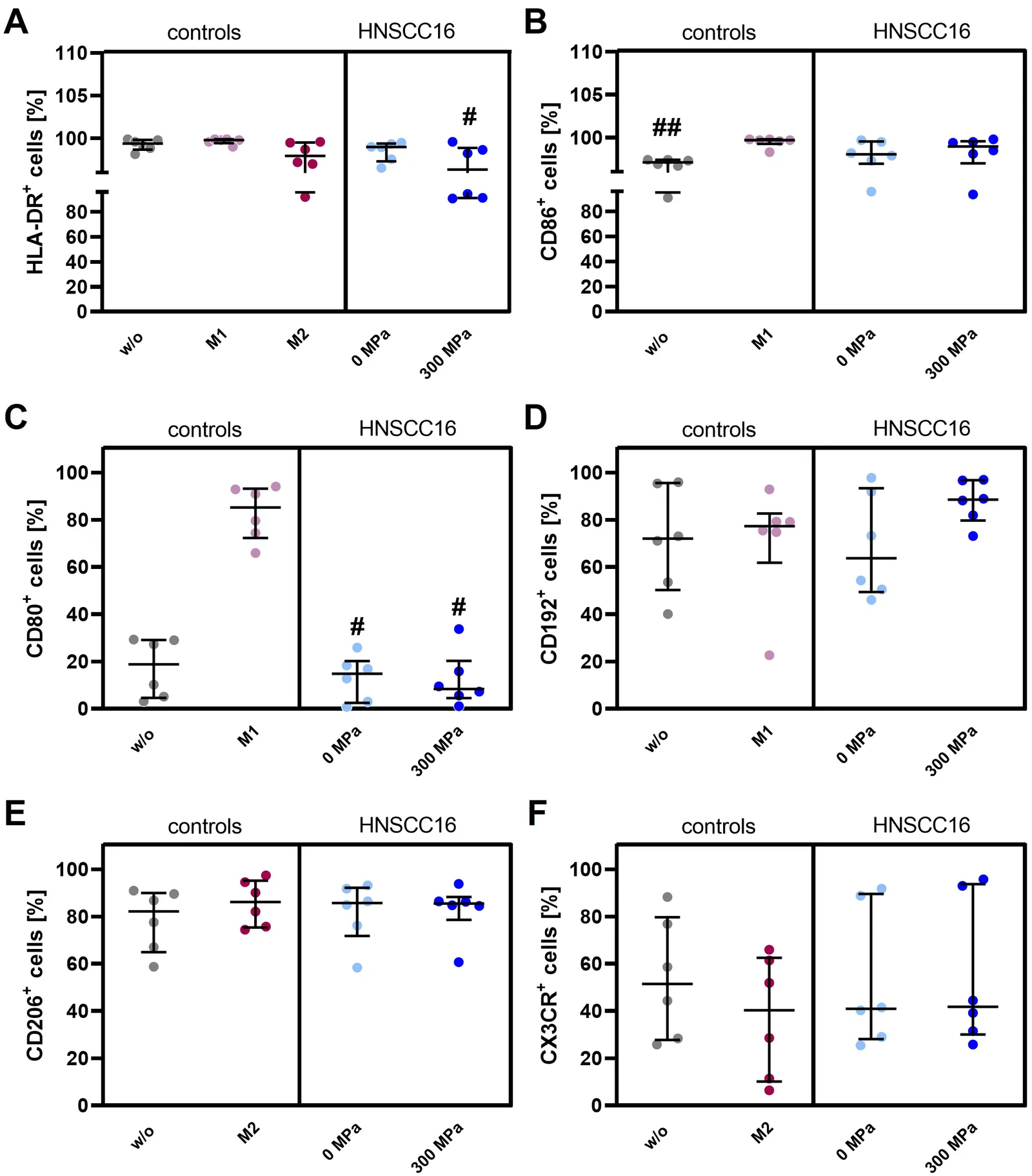
Flow cytometry data on the percentage of macrophages expressing polarization surface markers after three days of stimulation with high hydrostatic pressure (HHP)-conditioned medium show no statistically significant differences. Human macrophages were stimulated for 72 hours with conditioned media derived from untreated (0 MPa) or 300 MPa HHP-treated head and neck squamous cell cancer cells (HNSCC16). GM-CSF/LPS and M-CSF/IL-4 were used as positive controls for M1 and M2 macrophage polarization, respectively, while fresh medium served as an unstimulated control (w/o). Macrophages were harvested and stained with fluorochrome-conjugated antibodies against M1 macrophage markers HLA-DR **(A)**, CD86 **(B)**, CD80 **(C)**, CD192 **(D)**, and the M2 macrophage markers CD206 **(E)** and CX3CR **(F)**. Data are shown as median and interquartile range. Statistical significance was determined using the Kruskal-Wallis test with Dunn’s multiple comparisons test (n = 6 independent biological replicates; ^#^ vs. M1 control, p < 0.05; ^##^ vs. M1 control, p < 0.01).

HLA-DR surface expression was high across all experimental groups. A small but statistically significant reduction was observed between the percentages of HLA-DR positive cells of 300 MPa-CM treated cells (median: 96.35%) and M1 control cells (median: 99.80%; p = 0.0135) (**Figure 7A**). While M2 macrophage control cells (median: 97.95%) also showed a similar, lower median compared to the unstimulated samples (median: 99.40%), cells treated with 0 MPa-CM (median: 98.98%), and the M1 macrophage control (99.80%), these differences did not reach statistical significance.

The results for the M1 macrophage markers were inconclusive. While the number of CD86-positive cells (**Figure 7B**) following treatment with 300 MPa-CM (median: 98.95%) was similar to the level of the M1 macrophage control cells (median: 99.7%), which was significantly higher compared to the unstimulated samples (median: 97.10%; p = 0.0038), the number of CD80-positive cells (**Figure 7C**) treated with 300 MPa-CM (median: 8.365%, p = 0.0100 vs. M1 control) or 0 MPa-CM samples (median: 14.80%; p = 0.0115 vs. M1 control) were significantly reduced compared to the M1 control cells (median: 85.25%). While unstimulated samples also showed a low percentage of CD80-positive cells (median: 18.75%) compared to the M1 control samples, the differences were not statistically significant (p = 0.0607). At the same time, the proportion of CD192-positive cells (**Figure 7D**) in the samples stimulated with 300 MPa-CM (median: 88.55%) was higher than the proportion in the unstimulated (median: 72.05%), the 0 MPa-CM stimulated samples (median: 63.75%) or the M1 macrophage control (median: 77.25%). However, these differences were not statistically significant.

When examining the M2 marker CD206 (**Figure 7E**), positive cells were detected in all groups studied. Although no statistical differences were detected, the median of positive cells for the 300 MPa-CM samples (median: 85.50%) lies slightly below that of the M2 controls (median: 86.10%) and the 0 MPa-CM samples (median: 85.65), but higher than the median of the unstimulated samples (median: 82.20%). The median proportion of CX3CR-positive cells (**Figure 7F**) in the unstimulated cells (median: 51.45%) was higher than that of the other groups (M2 control: 40.25%; 0 MPa-CM: 40.90%; 300 MPa-CM: 41.80%). No statistical differences were found.

The flow-cytometry data demonstrate that HHP-treated HNSCC-derived CM alters selected macrophage surface markers, most notably HLA-DR, CD86, and CD80. However, the simultaneous presence of markers commonly associated with different activation states and the absence of coordinated changes across the marker panel further indicate that the response cannot be adequately described as a binary M1/M2 switch.

## 4 Discussion

New strategies are needed to address the high recurrence rate and the impact of tissue defects following extensive tumor resection in HNSCC (4). HHP treatment addresses the need for further improved therapeutic and regenerative strategies for HNSCC treatment (8, 13, 27–29). However, research concerning the immunological responses to tumor-derived mediators released by HHP treatment of tumor cells is limited but critically important both for the development of HHP-based whole-cell vaccines and the application of HHP in regenerative medicine (15).

The traditional classification of macrophages into M1 and M2 subtypes is limited. Macrophages are highly plastic and dynamically adjust their polarization state in response to changes in the cytokine environment. *In vivo*, they therefore exist as a spectrum of overlapping and potentially reversible activation states rather than as a distinct switch between M1 and M2 macrophages (17, 18, 21, 30). Nevertheless, the analysis of classic M1- and M2-like markers can still provide insight into the functions they may mediate. A pro-inflammatory, M1-like phenotype is important for early debridement of necrotic cells and clearance of microbes or residual tumor cells. However, persistent pro-inflammatory activity can impair graft vascularization and cause tissue damage (17–19). Alternatively activated M2 macrophages support tissue repair, angiogenesis, and resolution of inflammation (17–19, 30). However, tumor-associated macrophages (TAMs) with a M2 macrophage-like phenotype may be able to promote tumor progression and immune evasion if malignant cells are still present in the surrounding tissue (17–19). Therefore, this study aimed to investigate the extent to which conditioned medium of HHP-treated HNSCC cells influences macrophage polarization marker expression.

Whilst M1 macrophages are usually described as large, flattened, and round or irregularly shaped, M2 macrophages are usually described as elongated and spindle-shaped cells, with further differences described between M2 subtypes (31–33). Nonetheless, it has been shown that the mediators or cytokines involved in polarization can have varying effects on macrophage morphology and phenotype (18, 34). Since morphologically heterogeneous cells were observed in all experimental groups, it was not possible to draw conclusions about polarization based solely on morphology.

The HHP-amplitude-dependent increase in IL-1α and IL-1β in the 300 MPa-CM blanks, described in our previous study (16), is assumed to be related to the extent of cellular damage and the associated transition from apoptosis to necroptosis-like cell death that we observed when comparing 210 MPa and 315 MPa treatment of HNSCC cell lines (13). This transition from mostly apoptotic towards necroptosis-like depending on HHP amplitude has been reported across various cell types, like fibroblasts, osteoblasts, chondrocytes, and tumor cell lines (8, 12, 14, 35, 36). While necroptosis-like cell death generally leads to the release of IL-1α and biologically inactive pro-IL-1β, there is evidence that IL-1β can also be activated through caspase-1-independent mechanisms in sterile inflammation models (21, 37). Further DAMPs induced by HHP treatment include the translocation of calreticulin and the release of HMGB1 and ATP (8, 13). In the context of tissue regeneration, it is known that DAMPs released as a result of tissue injury recruit monocyte-derived macrophages to the wound site and are able to mediate sterile inflammation, anti-tumor immunity, or tissue repair (21, 38–40). Classically, M1 macrophages are activated upon binding of microbial products, such as LPS, to pattern recognition receptors (PRRs), which induce NF-κB-mediated expression of pro-inflammatory cytokines (17, 21, 30). In a similar way, DAMPs can bind to PRRs and promote the expression of M1 macrophage markers, including TNF-α, IL-6, IL-1β, IL-12, and IL-23 (17, 30, 38, 40). Similarly, IL-1 triggers signaling pathways via the IL-1 receptor that closely resemble those activated by PRRs, engaging the MyD88–IKK complex and ultimately activating NF-κB and MAPK pathways (21, 41). Both IL-1α and IL-1β have shown potential as adjuvants in vaccination against pathogens and for antitumor immunity by promoting inflammation and enhancing T-cell responses (42–44).

In our study, we could demonstrate that the stimulation with conditioned medium (CM) derived from 300 MPa-treated HNSCC cell lines significantly induced gene expression of IL-1β and IL-8, with a nonsignificant elevation in *IL6* gene expression compared to IL-4-stimulated M2 macrophage control cells. Except for IL-1β, these results were consistent with the protein analysis, and similar results were detected for the LPS-stimulated M1 macrophage control cells, supporting the presence of selected pro-inflammatory, M1 macrophage-like characteristics following stimulation with HNSCC-derived 300 MPa-CM. These findings are in accordance with the results of Moratal et al. (45) that showed an increased gene expression of M1 macrophage markers IL-6 and IL-1β in macrophages stimulated with IL-1β compared to unstimulated samples or macrophages stimulated with IL-4. Given that IL-1β is a driver of M1 macrophage-like polarization (38, 46, 47) and that it amplifies its own expression through autocrine feedback (46) while also promoting the JNK/c-Jun-dependent upregulation of pro-inflammatory mediators such as IL-6 and IL-8 (48), it is possible that IL-1β plays a central role in driving the observed cytokine profile, particularly the sustained expression of IL-1β and IL-8. Nevertheless, this proposed mechanism was not directly investigated in the present study and should therefore be considered hypothetical. Compared to CM blanks of untreated HNSCC cells, the IL-1β content was elevated in 300 MPa-CM blanks for both cell lines, and this may be the reason for the different results between the stimulation groups. Thus, the contribution of pre-existing IL-1β in the CM needs to be considered when interpreting the macrophage-derived IL-1β response.

While the gene expression analysis suggests a strong induction in *IL1B* expression, the high IL-1β protein content in the 300 MPa-CM blanks complicates the precise determination of macrophage-mediated IL-1β release. When IL-1β levels in the unincubated blanks were subtracted from those in the 72-hour stimulated samples, little to no detectable IL-1β remained for the 300 MPa-CM groups, suggesting that the signal largely originated from exogenous IL-1β rather than *de novo* cellular production. However, given the short half-life of IL-1β in serum (37, 49) and its susceptibility to degradation through oxidation, aggregation, and proteolytic cleavage by matrix metalloproteinases (MMPs) (50, 51), we conducted an additional control experiment to better distinguish between residual and newly produced IL-1β. To this end, all blanks were incubated under identical conditions (37 °C, 72 h) as the stimulated cultures to assess the persistence of the initial IL-1β pool. Following incubation, the IL-1β level in the blanks decreased by approximately a four- to sixfold. After subtracting these incubated blanks from the stimulated samples, IL-1β levels remained significantly elevated in 300 MPa-CM-treated samples compared to M2 macrophage control samples. This comparison is consistent with, but does not definitively demonstrate, *de novo* synthesis and secretion by macrophages in response to stimulation with 300 MPa-CM. However, several limitations should be considered. Although incubation of CM blanks under identical conditions accounts for cytokine decay, it does not fully recapitulate the cellular environment, where protease activity, cytokine binding, and uptake may differ. Moreover, while blanks contain a fixed initial IL-1β pool, stimulated samples reflect the net balance of continuous secretion and degradation over time. In addition, the protein-based readout does not distinguish between newly synthesized and residual or partially degraded IL-1β. Therefore, although the data is consistent with *de novo* IL-1β production, contributions from differential degradation dynamics cannot be entirely excluded. Additional approaches such as metabolic labeling or intracellular IL-1β measurements would be required to definitively distinguish newly synthesized form residual IL-1β.

Importantly, despite the induction of IL-8, IL-1β, and potential IL-6 following 300 MPa-CM stimulation, we observed no significant protein increase of TNF-α, IL-12 p70, or IL-23. This selective cytokine profile is consistent with the absence of co-stimulatory signals required for full M1 activation, suggesting that HHP-induced DAMPs may initiate a partial or context-specific M1-like response, but the underlying mechanism was not investigated in this study. Thus, rather than representing a conventional M1 phenotype, the observed cytokine profile is more appropriately interpreted as a partial M1-like or pro-inflammatory activation state. This nuanced activation could potentially be advantageous in regenerative settings, where excessive inflammation must be balanced with tissue repair. However, whether this response is beneficial for tissue regeneration cannot be determined from the present *in vitro* data and requires validation in appropriate tissue or *in vivo* models.

M2 macrophage-like TAMs are typically characterized by low expression of pro-inflammatory cytokines, especially IL-12, CXCL9, and CXCL10, but high levels of IL-10, and are often associated with tumor progression and metastasis by immune suppression (52, 53). While no significant differences of the protein content of CXCL10 or CXCL9 were observed, a trend toward increased CXCL9 secretion was detected in macrophages treated with 300 MPa-CM compared to M2 macrophage control samples. The chemokines CXCL9 and CXCL10 are involved in immune cell recruitment, including Th1 and cytotoxic T-cells, to the inflammation site and are associated with enhanced tumor suppression with improved therapeutic potential in different tumor entities, including ovarian and lung cancer (53–57). Therefore, the induction of CXCL9 secretion following stimulation with 300 MPa-CM could potentially contribute to anti-tumor responses against potentially still remaining cancer cells after reimplantation of the devitalized tissue in the former tumor site. This interpretation remains hypothetical, as clearance of residual tumor cells and anti-tumor activity were not directly assessed in the present study. However, high CXCL9 expression may indicate strong inflammation, negatively affecting graft healing. That is why the early detection of urinary CXCL9 and CXCL10 levels is investigated for its use as biomarkers for kidney allograft rejection (58). Since elevated numbers of CX3CR1-positive macrophages are associated with TAMs as well as T-cell-mediated allograft rejections, we further investigate the potential of 300 MPa-CM-treated macrophages to influence the expression of CX3CR1 (17, 59). Compared to untreated cells and macrophages stimulated with 0 MPa-CM, no significant changes in CX3CR1 expression were observed for 300 MPa-treated macrophages. However, given that CX3CR1 expression alone does not directly establish the potential for graft rejection, further *in vivo* studies are required to determine whether the altered mediator profile induced by HHP-treatment of HNSCC cells effects T-cell-mediated responses and the integration of HHP-treated tissue grafts.

We observed no significant induction of the M2 macrophage markers IL-10, MMP-9 (30, 60) or the chemokines CCL17, CCL18, and CCL22 (30, 61) at either the gene or protein level following stimulation with 0 MPa- or 300 MPa-CM, further suggesting an induction toward a rather pro-inflammatory, M1 macrophage-like polarization. However, the absence of these marker induction cannot exclude the possibility of intermediate or mixed activation states that were not detected by the selected panel of markers. Notably, however, none of the observed stimulation groups showed an increased expression of the analyzed M2 macrophage markers compared to unstimulated cells, not even the M-CSF and IL-4-treated M2 macrophage control samples. These findings are in contrast to the results of Moratal et al. (45), who observed an increased CCL18 gene expression in IL-4 polarized macrophages, as well as to the results of Huang et al. (61) who demonstrated significantly enhanced protein synthesis rates of IL-10, CCL17, CCL18, and CCL22 in IL-4 stimulated THP-1 cells. In contrast to the findings of this study, cancer cell-derived conditioned media of melanoma (62), breast adenocarcinoma (59), and colon cancer (63) influenced macrophage polarization towards a M2 macrophage-like phenotype. This resulted in the expression of several M2 macrophage markers, including IL-10, CCL17, CCL22, and CD206, while simultaneously expressing M1 markers, e. g., TNF-α or IL-6. This highlights the existence of mixed or hybrid activation states in TAMs rather than conventional M1 or M2 macrophage polarization (64, 65).

Our results demonstrate that CM from HNSCC cells treated with 300 MPa induces an M1-like macrophage phenotype that differs from classical M1 polarization. This assumption is further supported by the high proportion of macrophages expressing the M1 macrophage surface markers CD86 (18), and CD192 (20, 33) following stimulation with this CM. The percentage of CD86-expressing macrophages was comparable to the M1 control, which showed a statistically significant increase in CD86-expressing cells compared to the unstimulated control. In contrast to the M1 macrophage control, the relative number of cells expressing CD80 was decreased in CM-treated cells, regardless of HHP treatment. Although both CD80 and CD86 are commonly associated with pro-inflammatory macrophage activation, their expression is regulated in a context- and stimulus-dependent manner. CD86 is rapidly induced following inflammatory activation, whereas CD80 expression depends on the extent and type of the activating stimulus (66–68). Therefore, the differential expression of CD80 and CD86 observed in this study may reflect a stimulus-specific activation rather than an inconsistency between individual polarization markers. This interpretation is in line with the current understanding that macrophage activation represents a continuum of functional states shaped by the surrounding microenvironment rather than discrete M1 and M2 phenotypes (18). Thus, the differential expression of these markers may be indicative of a stimulus-specific activation state. While CD86 and high HLA-DR expression are typical M1 macrophage maker, they are also characteristic of M2b macrophages, which are defined by low CD80 expression, the production of the pro-inflammatory cytokines IL-1β, IL-6 and TNF-α, and low IL-12 p70 levels (18, 19, 30, 69). However, M2b macrophages secrete high levels of the anti-inflammatory marker IL-10 (18, 19, 30) that was not induced at either the gene or protein level following CM stimulation in this study. Although CD206 expression was high in the 300 MPa-CM group, it was comparable to that in the unstimulated and 0 MPa-CM controls. The concurrent expression of CD86 and CD206 across all groups may partly reflect baseline culture conditions rather than specific CM stimulation. Overall, CM from HHP-treated HNSCC cells was associated with a partially activated M1-like macrophage phenotype under the conditions investigated. Importantly, the present data do not support assignment to a defined macrophage subtype, but rather indicate a macrophage phenotype with selected pro-inflammatory characteristics.

## 5 Conclusion

Our present study demonstrates that conditioned media from HHP-treated HNSCC cells induces a partially activated macrophage state distinct from classical M1 or M2 macrophage polarization phenotypes. The observed profile, characterized by elevated pro-inflammatory markers and expression of typical M1 surface markers, but lacking CD80 expression, TNF-α, and IL-10, is consistent with a stimulus-specific and pro-inflammatory activation state rather than a fully polarized M1 phenotype. The concurrent retention of M2-associated markers (CD206, CX3CR1) across all groups indicates that this mixed profile may be influenced by baseline culture conditions, but does not establish a defined M2-like macrophage phenotype. These findings demonstrate that while HHP treatment alters the cytokine milieu of tumor-derived factors, the resulting immune response does not appear in order to drive an immunosuppressive state. Consequently, HHP-devitalized autologous cartilage tissue may represent a potential candidate for orthotopic reconstruction, though further *in vivo* studies are necessary to validate these immunological interactions within the complex tissue microenvironment. In particular, future studies should determine whether the observed partial pro-inflammatory response contributes to the clearance of residual tumor cells without compromising graft integration and tissue regeneration.

### 5.1 Limitations

This study has several limitations. While *in vitro* two-dimensional cell cultures provide an initial means of investigating macrophage behavior in response to tumor cell-derived factors, they cannot fully replicate the complex *in vivo* microenvironment, including the interactions between multiple immune cell populations, stromal cells, and extracellular matrix components. We therefore emphasize this limitation and acknowledge that validation in more physiologically relevant systems, such as three-dimensional tumor models and appropriate *in vivo* models, will be essential to confirm the translational relevance of our findings. Although macrophage activation was characterized by complementary analyses of gene expression, cytokine secretion, and surface marker expression, additional functional assays, such as phagocytosis, T-cell activation, or tumor cell killing assays, are required to determine the biological relevance of the observed phenotype. Furthermore, future work should investigate the contribution of individual cytokines and damage-associated molecular patterns released following HHP treatment to further elucidate the underlying mechanisms. The contribution of residual mediators in the CM, particularly IL-1β, also requires further investigation using experimental approaches that can distinguish exogenous from newly synthesized protein. To better assess the immunological response to HHP-treated cartilage tissue intended for orthotopic reimplantation following tumor resection, future experiments should utilize autologous, superficially tumor-infiltrated tissue.

## Data Availability

The raw data supporting the conclusions of this article will be made available by the authors, without undue reservation.
