# Supplementary material for: Immunomodulatory effects of conditioned medium from high hydrostatic pressure-treated head and neck squamous cell carcinoma cells regarding macrophage polarization
Source: Front Immunol. 2026 Oct 19;17:1885288. doi: 10.3389/fimmu.2026.1885288 (PMC13724621; doi:10.3389/fimmu.2026.1885288)
Supplement: Supplementary file 1 [file Table1.docx]

Supplementary Material

**Figure 1** Actin/DAPI staining of adherent mononuclear cells after 24 hours of stimulation with conditioned medium (CM) derived from untreated (0 MPa; E, G) and high hydrostatic pressure (HHP) treated (300 MPa; F, H) head and neck squamous cell cancer cell lines HNSCC16 (E, F) or UTSCC14 (G, H). Positive controls for M1 macrophage polarization (GM-CSF & LPS; C) and M2 macrophage polarization (M-CSF & IL-4; D) were used, while fresh medium (w/o stimulation; A) and CM of HHP treated chondrocytes (B) served as negative controls. The actin cytoskeleton of the cells was stained with phalloidin (green fluorescence). The nuclei were counterstained with DAPI (blue fluorescence). Fluorescence images were acquired using a Keyence BZ -X810 fluorescence microscope and combined using Keyence BZ-X800 Analyzer Software to generate composite fluorescence images (scale bar 50 µm).

Figure 2 Flow cytometry gating strategy for the M1 (A) macrophage polarization panel and the M2 (B) macrophage polarization panel used in this study. Representative results are shown for macrophages stimulated for 72 hours with conditioned medium derived from the high hydrostatic pressure-treated head and neck squamous cell carcinoma cell line HNSCC16.

Table 1 Target protein concentration in the respective blanks

| **Target protein [pg/ml]** | **Fresh medium blank** | **0 MPa HNSCC16 CM blank** | **300 MPa HNSCC16 CM blank** | **0 MPa UTSCC14 CM blank** | **300 MPa UTSCC14 CM blank** |
| --- | --- | --- | --- | --- | --- |
| **CCL17** | 101.43 | 94.32 | 115.51 | 115.51 | 101.43 |
| **CCL18** | n. d. | n. d. | n. d. | n. d. | n. d. |
| **CCL22** | 77.32 | 80.24 | 80.89 | 77.642 | 77.97 |
| **CXCL9** | 703.71 | 684.91 | 722.4 | 777.84 | 759.46 |
| **CXCL10** | n. d. | n. d. | n. d. | 11.11 | 6.53 |
| **IL-1β _Luminex_** | n. d. | n. d. | 139.11 | 25.33 | 679.40 |
| **IL-1β _0 h_** | 0.0244 | 3.82 | 115 | 15.8 | 739 |
| **IL-1β _72 h_** | 0.503 | 0.61 | 28.6 | 3.89 | 143 |
| **IL-6** | n. d. | 10.38 | 3.05 | 12.09 | 4.17 |
| **IL-8** | n. d. | 44.52 | 10.21 | 488.10 | 237.76 |
| **IL-10** | n. d. | 29.48 | n. d. | n. d. | n. d. |
| **IL-12 p70** | 0.09 | 18.63 | n. d. | 0.09 | 0.09 |
| **IL-23** | n. d. | 112.41 | n. d. | 281.62 | 207.89 |
| **MMP-9** | 359.96 | n. d. | n. d. | 345.86 | 184.23 |
| **TNF-α** | n. d. | 112.41 | n. d. | 281.62 | 207.89 |
